# Assessment of Lewis‐Acidic Surface Sites Using Tetrahydrofuran as a Suitable and Smart Probe Molecule

**DOI:** 10.1002/open.202200021

**Published:** 2022-03-24

**Authors:** Samih A. Halawy, Ahmed I. Osman, Adel Abdelkader, Mahmoud Nasr, David W. Rooney

**Affiliations:** ^1^ Nanocomposite Catalysts Lab. Chemistry Department Faculty of Science at Qena South Valley University Qena 83523 Egypt; ^2^ School of Chemistry and Chemical Engineering Queen's University Belfast David Keir Building Belfast BT9 5AG UK

**Keywords:** acidity, Lewis acid, pyridine, THF, temperature-programmed desorption

## Abstract

Measuring the Lewis‐acidic surface sites in catalysis is problematic when the material‘s surface area is very low (S_BET_ ≤1 m^2^ ⋅ g^−1^). For the first time, a quantitative assessment of total acidic surface sites of very small surface area catalysts (MoO_3_ as pure and mixed with 5–30 % CdO (wt/wt), as well as CdO for comparison) was performed using a smart new probe molecule, tetrahydrofuran (THF). The results were nearly identical compared to using another commonly used probe molecule, pyridine. This audition is based on the limited values of the surface area of these samples that likely require a relatively moderate basic molecule as THF with p*K*_b_=16.08, rather than strong basic molecules such as NH_3_ (p*K*_b_=4.75) or pyridine (p*K*_b_=8.77). We propose mechanisms for the interaction of vapour phase molecules of THF with the Lewis‐cationic Mo and Cd atoms of these catalysts. Besides, dehydration of isopropyl alcohol was used as a probe reaction to investigate the catalytic activity of these catalysts to further support our findings in the case of THF in a temperature range of 175–300 °C. A good agreement between the obtained data of sample MoO_3_‐10 % CdO, which is characterised by the highest surface area value, the population of Lewis‐acidic sites and % selectivity of propylene at all the applied reaction temperatures was found.

## Graphical abstract

**Measuring Lewis‐acidic surface sites** in catalysis is problematic when the material‘s surface area is very low (S_BET_ ≤1 m^2^ ⋅ g^−1^). For the first time, a quantitative assessment of total acidic surface sites of small surface area catalysts was performed using a new probe molecule, tetrahydrofuran. The results were nearly identical when compared to other commonly used probe molecule, pyridine. This work may be considered ground‐breaking in this field by applying the temperature‐programmed desorption (TPD) technique using both thermogravimetric (TG) and differential scanning calorimetry (DSC) analyses.

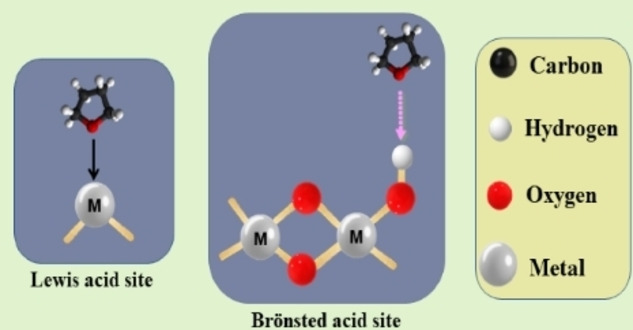

## Introduction

In the last seven decades, great progress has been made in the acidity measurements of solid catalysts. Many researchers have estimated qualitatively and/or quantitatively‐semi‐quantitatively the acidic surface sites, particularly the external Brönsted acid sites, over a wide range of solid catalysts with high surface area and large pore volumes such as zeolites, alumina and SiO_2_[^1^, ^2^] by using some compounds rather than the most common molecules, that is, NH_3_ and pyridine. Some of these attempts failed, while others yielded acceptable and successful results.[^1^, ^2^] The majority of the published articles employed the IR‐spectroscopic methods to characterise the surface acidity of solid catalysts such as: 12‐membered ring zeolites probed with 2,4,6‐tri‐*tert*‐butylpyridine,^[1]^ TiO_2_‐based solid catalysts by adsorption of NH_3_,^[2]^ commercial samples of Faujasite‐type zeolite probed with six molecules as follows: pyridine, 2,6‐di‐*tert*‐butylpyridine, 2,4 dimethylquinoline, tributylamine, trihexylamine, and 2,4,6‐tri‐*tert*‐butylpyridine that with different basicities and critical diameters.^[3]^ Others demonstrated the NH_3_‐TPD (Temperature‐programmed desorption) profiles of Fe_2_O_3_ nanoparticles supported on γ‐χ‐Al_2_O_3_,^[4]^ and promoted Mg−Al mixed oxides with transition metals.^[5]^ Gafurov et al.^[6]^ employed electron paramagnetic resonance (EPR) measurements of 9,10‐anthraquinone as a probe molecule, as well as IR spectroscopy of CO, pyridine and NH_3_‐TPD to successfully determine and differentiate between the strength of the surface Lewis acid centres of γ‐alumina. They observed a good agreement between the EPR results of anthraquinone and the other exploited methods.^[6]^ Many recent research articles explained the use of a new technique, probe‐assisted nuclear magnetic resonance (NMR), to investigate the catalyst‘s surface of any solid acid catalysts, based on the chemical shift of a given probe molecule such as trimethylphosphine (TMP).[^7^, ^8^, ^9^, ^10^, ^11^] This advanced technique provides qualitative evidence (including both types and distribution of these acidic sites, as well as their basic interactions) besides the quantitative information on all acid sites, that is, strength and populations.

The above‐mentioned successful attempts,[^1^, ^2^, ^3^, ^4^, ^5^, ^6^, ^7^] which used different basic molecules, encourage us to investigate tetrahydrofuran (THF) as a probe molecule to determine acidic surface sites of our samples. Our research team has published numerous articles over the last two decades, including TPD studies of pyridine and NH_3_ as probe molecules, as well as IR‐spectroscopy of pyridine[^12^, ^13^, ^14^, ^15^, ^16^] for the determination of the acidic sites of some catalysts.

The innovation in the current study is the successful quantitative assessment of the total acidic surface sites of samples with very low surface area, using a smart new probe molecule THF. This work may be considered ground‐breaking in this field by applying the TPD technique using both TG and DSC analyses. Our findings were compared with those obtained using another widely used probe molecule, that is, pyridine, and the results were almost identical.

## Experimental Section

### Catalyst Preparation

CdO and MoO_3_ were obtained by direct calcination of Cd(NO_3_)_2_ ⋅ 4H_2_O (BDH, England) and ammonium heptamolybdate (NH_4_)_6_Mo_7_O_24_ ⋅ 4H_2_O (AHM) (Fisons, England) at 500 °C for 5 h in static air. Also, three samples of MoO_3_ mixed with 5, 10 and 30 mol % CdO were prepared as follow: calculated amounts of Cd(NO_3_)_2_ ⋅ 4H_2_O (i. e. 1.54, 3,08 and 9.24 g) were dissolved in 10 mL of deionised water, then the corresponding weights of AHM (as 16.77, 15.89 and 12.36 g) were suspended in the solution. The mixtures were then evaporated to dryness over a water bath, with continuous stirring. The resulting materials were dried at 120 °C and then calcined in static air for 5 h at 500 °C.

### Characterisation

Powder X‐ray diffraction (XRD) of the catalysts were carried out using a Siemens D5000 (Germany) at ambient temperature. The instrument used Ni‐filtered CuK_α_ radiation (λ=1.5418 Å) an electron source of 40 kV and 30 mA. Diffractograms were recorded for 2θ values ranging from 10 to 70° with a scanning speed of 0.01 s per step. The diffraction patterns thus obtained were compared with references from the JCPDS database to identify phases and characterise materials.

Temperature‐programmed reduction (TPR) of the catalyst samples calcined at 500 °C was performed using a conventional apparatus,^[17]^ consisting of a gas supply system with mass‐flow controller, a quartz U‐reactor, a water vapour trap and a thermal conductivity detector (TCD). The sample bed temperature was monitored with a thermocouple protected by a quartz tube inserted in the centre of the sample bed with its tips located a few millimetres above the sample bed. A sample weight of ≈10 mg was always used, and H_2_ consumption peak was monitored by thermal conductivity detector while the sample was heated from ambient to 1000 °C at a heating rate of 5 °C ⋅ min^−1^ in a stream 6 % H_2_/N_2_ (40 mL ⋅ min^−1^) gas mixture with a purity of 99.999 %. In order to minimise the contribution of adsorbed species to TPR profiles, prior to TPR experiments, all samples were preheated in N_2_ at 150 °C for 1 h.

Surface area measurements were carried out at liquid nitrogen temperature (−196 °C), with an automatic ASAP 2010 Micromeritics sorptometer (USA). Before each measurement, the samples (500 mg) were degassed at 200 °C and 10^−5^ Torr for 3 h (1 Torr=133.3 Pa). The surface area was calculated according to the BET method applying 5‐points programme for low surface area using nitrogen.

The total number of acidic sites (sites ⋅ g^−1^) of each sample was measured using the temperature‐programmed desorption (TPD) of pyridine (Pyr) and tetrahydrofuran (THF, 99+%, stabilised with 0.025 % butylated hydroxytoluene‐Sigma) condensed phase, as probe molecules, in two separate experiments for comparison. The experimental details have been described previously.[^15^, ^16^] This was achieved using 50 mg of sample preheated at 350 °C for 1 h in air before exposure to the probe molecule. 20 ± 2 mg covered sample with Pyr or THF were subjected to thermogravimetric analysis (TGA) and differential scanning calorimetry (DSC) analyses at a heating rate of (10 °C ⋅ min^−1^) in dry N_2_ flowing (40 mL ⋅ min^−1^), using a 50H Shimadzu thermal analyser (Japan). The thermal analyser is equipped with a data acquisition and handling system (TA‐50WSI). α–Al_2_O_3_ was used as the reference material in DSC measurements. The mass loss due to the desorption of Pyr or THF during TG experiments from the acidic sites was determined to measure the total surface acidity as sites ⋅ g^−1^. The following equation is used to estimate the total number of acidic surface sites as in Equation 1:^[15]^


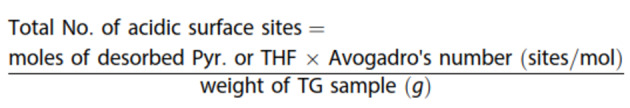




**Scheme 1:**
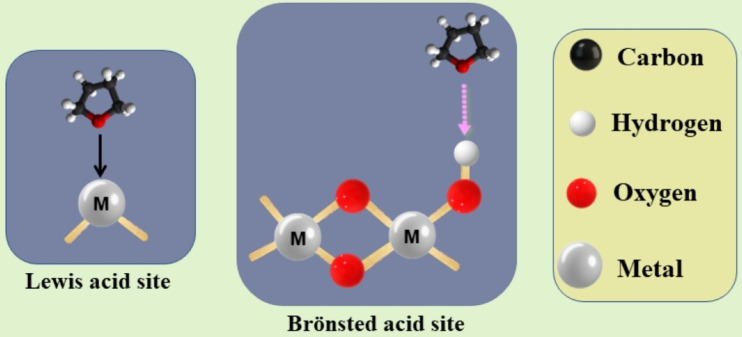
Interaction modes of THF vapour molecules with Lewis and Brönsted acid sites.

In order to further support our results concerning the surface acidity of samples under study, we used THF as a probe molecule. Scheme 1 gives general form of the adsorption modes of THF with Lewis and Bronsted sites. The dehydration reaction of isopropyl alcohol (IPA)[^18^, ^19^] was carried out over these samples in a fixed‐bed reactor with a continuous flow system under atmospheric pressure as follows: 200 mg of each sample was used and stabilised in a stream of dry N_2_ (in 1 cm i.d. pyrex glass reactor and 16.5 cm long) for 1 h at 400 °C before measurements. Dry N_2_ with a flow rate of 150 mL ⋅ min^−1^ was passed through liquid IPA at 5 °C. The reactor effluent during the catalytic reaction was analysed by a gas chromatograph (Shimadzu GC‐14A) equipped with a data processor model Shimadzu Chromatopac C−R4AD (Japan). A flame ionisation detector (FID) and a stainless‐steel column (PEG 20 M 20 % on chromosorb W, 60/80 mesh, 3 mm×3 mm i.d.) at 110 °C were used.

## Results and Discussion

### X‐Ray Diffraction Measurements

**Figure 1:**
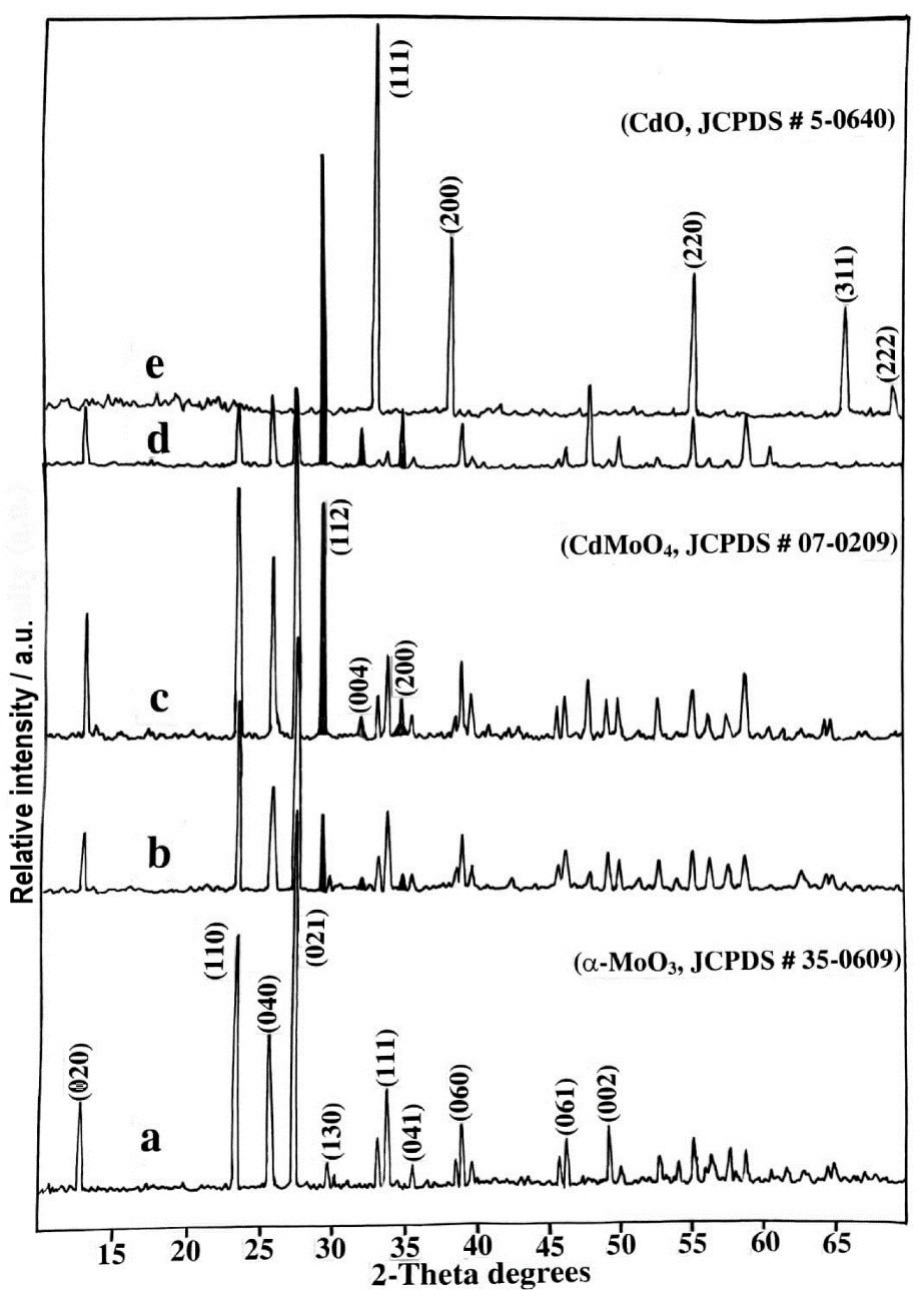
XRD patterns of pure MoO_3_ (a), CdO (e) and MoO_3_ mixed with 5–30 % CdO (b‐d) calcined at 500 °C for 5 h in air.

XRD measurements have demonstrated the well‐crystalline structure of the five prepared catalysts, that is, MoO_3_, CdO and MoO_3_ mixed with 5, 10 and 30 % CdO, that were calcined at 500 °C for 5 h, as nanoparticles, see Figures 1a–e. All patterns of pure MoO_3_ and mixed oxide samples a‐d showed a group of sharp diffractions of orthorhombic MoO_3_ (α‐MoO_3_, JCPDS#35‐0609) at 2θ=12.77, 23.31, 25.75, 27.30, 29.43, 33.75, 35.45, 39.32, 46.51 and 49.37° which correspond to (020), (110), (040), (021), (130), (111), (041), (060), (061) and (002) crystal facets (α‐MoO_3_, JCPDS#35‐0609).[^20^, ^21^] On the other hand, the XRD pattern of CdO clearly displayed five very strong diffraction peaks at 2θ=33.07° (111), 38.34° (200), 55.30° (220), 65.96° (311) and 69.26° (222) which belong to face‐centred system (Monteponite CdO, JCPDS # 5‐0640) as shown in Figure 1e.[^22^, ^23^] Three distinct diffraction peaks can be observed in patterns b‐d of mixed MoO_3_‐CdO samples at 2θ=29.25° (112), 31.99° (004) and 34.38° (200), which could be related to the formation of tetragonal phase CdMoO_4_ in these mixtures (JCPDS # 07–0209).[^24^, ^25^, ^26^] The crystallite size of the obtained different phases in these samples, that is, α‐MoO_3_, CdO and CdMoO_4_, was calculated using the Scherrer equation.^[22]^ For MoO_3_ in all patterns a‐d, the full width at half maximum (FWHM) of the narrow Debye–Scherrer line at 2θ=27.30° was used to calculate the crystallite size of MoO_3_, revealing a size range of 65–80 nm.^[27]^ Furthermore, the diffraction line at 2θ=29.25° was used for calculations in the case of tetragonal CdMoO_4_,^[26]^ where its crystallite size was calculated to be in the range of 69–82 nm. Finally, two diffraction lines at 2θ=33.07° and 38.34° were used to calculate the crystal size of CdO “Monteponite” (JCPDS # 5–0640) where a value between 44–66 nm was found for the crystallite size of CdO.^[28]^ The XRD results of the formation of CdO and CdMoO_4_ in the nanoscale, further, are consistent with previously published articles.[^29^, ^30^]

### Temperature‐Programmed Reduction (TPR)

**Figure 2:**
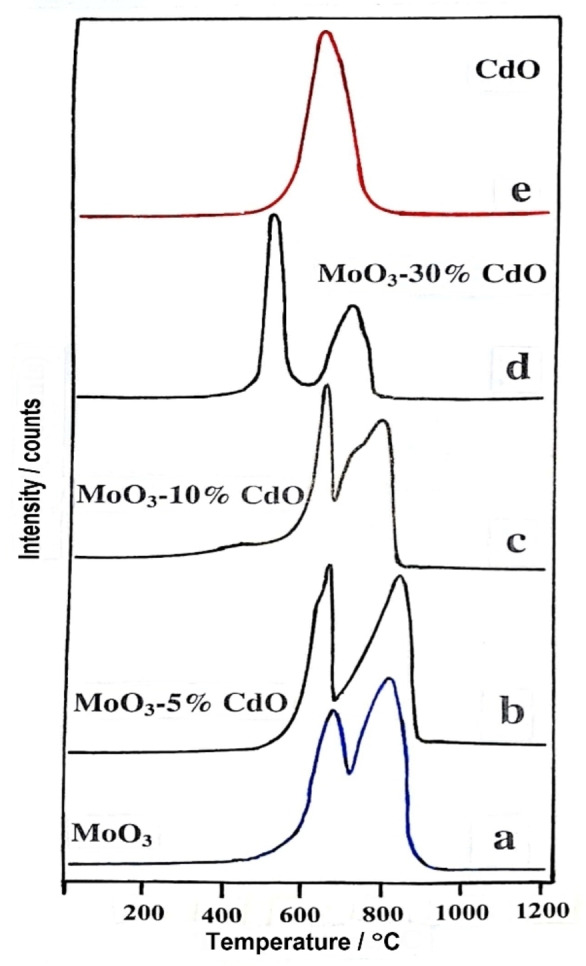
TPR profiles for pure: MoO_3_ (a), CdO (e) and MoO_3_ mixed with 5–30 % CdO (b‐d), calcined at 500 °C for 5 h in air.

**Scheme 2:**
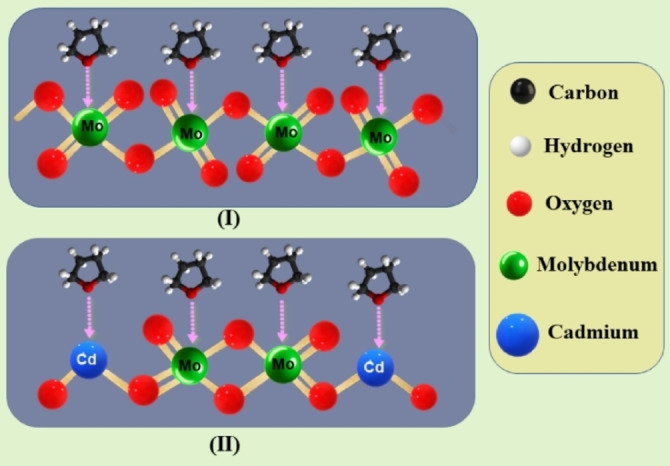
(I) Interaction of THF vapour molecules with Lewis‐acidic Mo cationic centres of pure MoO_3_. (II): Interaction of THF vapour molecules with Lewis‐acidic Mo and Cd‐cationic centres of CdMoO_4_.

TPR experiments are carried out to obtain information about solid materials (i. e., oxides and mixed metal oxides) that are often used in the field of heterogeneous catalysis. The reduction profiles of MoO_3_‐CdO samples, calcined at 500 °C in air for 5 h, are presented in Figure 2. The TPR profile of MoO_3_ (Figure 2a) shows two major reduction peaks with T_max_ at 683 and 818 °C, corresponding to the reduction of MoO_3_ to MoO_2_, then MoO_2_ to Mo metal.^[31–34]^ On the other hand, the TPR profile of CdO (Figure 2e) exhibits a single peak at T_max_ **=**638 °C, attributed to the reduction of CdO to Cd metal.[^35^, ^36^] The addition of 5 mol % CdO to MoO_3_ facilitated the reduction of Mo^6+^ to Mo^4+^, where T_max_ of the first peak was lowered and appeared at 662 °C rather than 683 °C, while it retarded the reduction of Mo^4+^ to Mo metal as T_max_ of the second peak appeared at a higher temperature, that is, 843 °C instead of 818 °C. This may be attributed to the beginning of CdMoO_4_ formation in this sample, as shown by XRD analysis, see above. In addition, the amount of hydrogen required to convert MoO_3_ to Mo‐metal through MoO_2_ was equal to 2.19**×**10^−2^ mol ⋅ g^−1^. Adding 5 mol % CdO (wt/wt) to MoO_3_ slightly reduced hydrogen uptake to 2.12**×**10^−2^ mol ⋅ g^−1^. Increasing the (x) mol % CdO added to 10 and 30 % resulted in a significant decrease in the reduction temperatures with the appearance of a shoulder at 720 °C, Figure 2c, which became a sharp peak in the case of the sample containing 30 mol % CdO, Figure 2d. Therefore, a continuous decrease in hydrogen uptake values was observed, as presented in Table 1, whereas that for CdO was equal to 0.86**×**10^−2^ mol ⋅ g^−1^. The hydrogen uptake of our samples, as shown in Table 1, and the corresponding T_max_ of each sample coincide with each other and clearly explain the facilitating effect of CdO in the course of reduction of MoO_3_ in TPR experiments during the formation of CdMoO_4_ in the sample of MoO_3_‐30 mol % CdO, see the XRD pattern Figure 1d. The TPR profiles of all samples calcined at 500 C° for 5 h proved the presence of molybdenum and cadmium, whether as pure or in mixed samples, with oxidation states of Mo^6+^ and Cd^2+^, respectively, see Figure 2. Consequently, these oxidation states are clearly presented in the proposed mechanism (Scheme 2).

**Table 1:** Values of T_max_, H_2_ uptake of TPR, S_BET_ and data of acidity measurements (as weight loss or number of acidic sites) using THF and pyridine as probe molecules for MoO_3_, CdO and mixed samples calcined at 500 °C for 5 h in air.

Sample	TPR *T_max_* [ °C]	TPR H_2_ uptake [10^−2^ mol ⋅ g^−1^]	S_BET_ [m^2^ ⋅ g^−1^]	THF results		Pyridine results	
				*ML_THF_ mg ⋅ g^−1^	Total no. sites ⋅ g^−1^	*ML_Pyrid_ mg ⋅ g^−1^	Total no. sites ⋅ g^−1^
Pure MoO_3_	683, 818	2.19	0.58 ± 0.01	186.7	15.6×10^20^	131.6	10.0×10^20^
MoO_3_‐5 % CdO	662, 843	2.12	0.95 ± 0.02	181.7	15.2×10^20^	114.3	8.7×10^20^
MoO_3_‐10 % CdO	648, 781	1.13	1.02 ± 0.05	284.3	23.7×10^20^	115.0	8.8×10^20^
MoO_3_‐30 % CdO	514, 719	1.03	0.95 ± 0.03	194.6	16.2×10^20^	97.6	7.4×10^20^
Pure CdO	638	0.86	0.40 ± 0.04	172.9	14.4×10^20^	10.5	0.8×10^20^

*(Mass loss calculated from TG−TPD experiments,in the range of 125–450 °C).

### Acidity Measurements by Temperature‐Programmed Desorption Using TG and DSC Techniques

**Figure 3:**
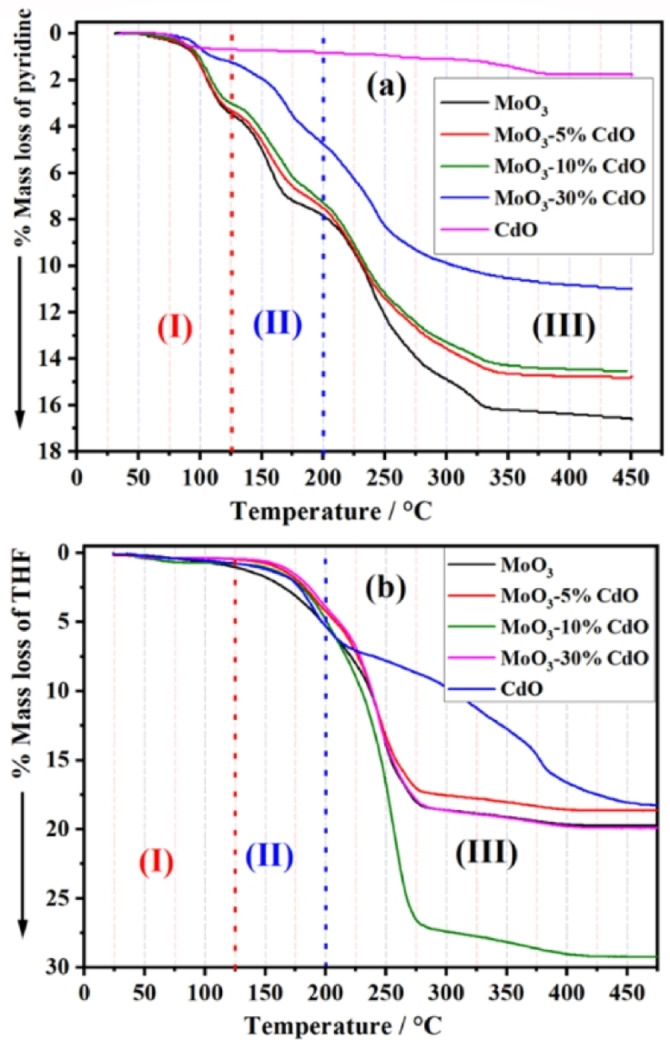
TG‐TPD curves of the % mass loss of pyridine (a) and THF (b) as probe molecules for samples calcined at 500 °C.

Herein, we will explain the experimental work concerning measurements of the TG and DSC‐TPD profiles using a new smart and promising probe molecule, tetrahydrofuran (THF), for the acidic sites of solid oxides, particularly those with very low surface area. Due to its popularity in this field, we first use pyridine (Pyr) as a probe molecule to distinguish between the strength of different acidic surface sites of our samples. Figure 3a demonstrates the TG‐TPD curves of the mass loss % due to desorption of Pyr‐molecules during heating samples from room temperature (RT) up to 475 °C. These curves monitor three mass loss zones^[37]^ in the following temperature ranges as: zone‐I (RT‐125 °C) due to desorption of physisorbed Pyr molecules, Zone‐II (125‐200 °C) ascribed to desorption of Pyr molecules from weak acidic sites, while the third zone (200‐450 °C) is corresponding to the Pyr desorption from moderate and strong acidic sites of these samples.

The total % mass loss at 450 °C due to desorption of Pyr‐molecules decreased as the (x) mol % of CdO added to MoO_3_ increased. MoO_3_ had the highest percent mass loss value, which was 16.53 %. CdO, on the other hand, had the lowest value of % mass loss of 1.7 %. The weights of Pyr. and THF (in mg/g_**solid**_) were calculated from TG‐TPD curves, Figures 3a,b, and are shown in Table 1. By examining the calculated quantity of both Pyr and THF whether (in mg/g_**solid**_) or (no. of acidic sites/g_**solid**_), the following points should be noted: ‐

1. In the case of Pyr, MoO_3_ had the highest values of acidic sites, while the addition of CdO gradually decreased the acidity, that is, from 10.0×10^20^ sites g^−1^ to 7.4×10^20^ sites g^−1^ in case of MoO_3_‐30 mol % CdO, see Table 1. The values mentioned above do not match the measured surface area of these samples due to their very low surface area.
2. On the other hand, TG‐TPD curves of THF showed the same three zones as pyridine. Except for CdO, the calculated values for THF as a probe molecule were always higher and approximately 1.6‐2.7 times those of Pyr (see Table 1). This unusual value of desorbed THF‐molecules, in the case of CdO, may be false and could be related to a special or other interaction between CdO and THF molecules, unrelated to acidic site desorption. More experiments with different tools and techniques will be required in the near future to clarify this particular situation. This discrepancy between calculated values of THF and pyridine, in this study, was similarly early noticed and published^[38]^ when applying NH_3_ and pyridine as probe molecules for the determination of numbers of Lewis and Brönsted acidic sites.
3. THF results, as shown in Table 1 and Figure 4
  **Figure 4:**
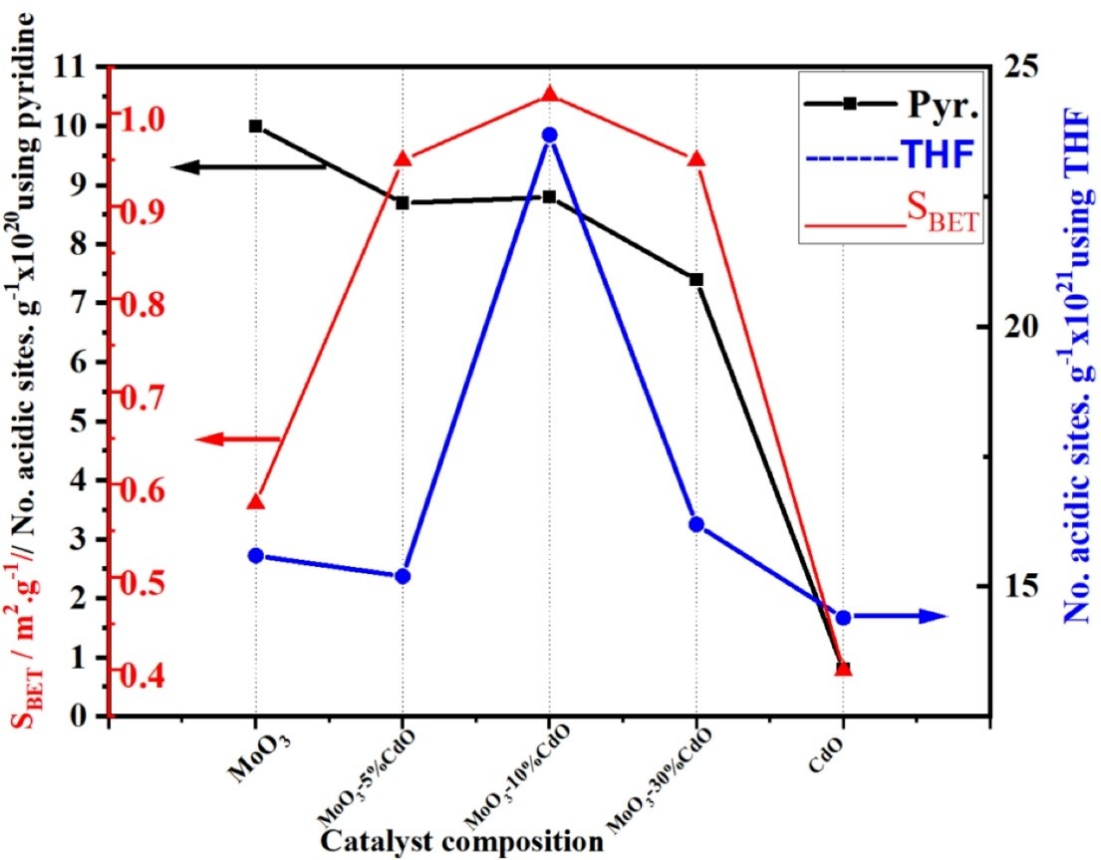
Variations of surface area and the total number of acidic sites, probing pyridine and THF molecules, as a function of catalysts composition.
  , are in good agreement with their very low surface area. The surface area (S_BET_) of our samples, whether MoO_3_ as pure or mixed with 5–30 mol % CdO (wt/wt), agrees with those published in recent works of literature[^31^, ^39^] in the range of 2.5‐0.7 m^2^ ⋅ g^−1^. This is an advantage when using THF as a probe molecule in our successful attempt. In addition, the most active sample during the dehydration reaction of IPA to propylene, that is, MoO_3_‐10 %CdO, as will be presented in Figure 8, gained the highest population of acidic surface sites besides the highest surface area (Table 1 and Figure 4). Its highest catalytic activity is attributed to these acidic sites^[18]^. This supports our hypothesis that THF outperforms pyridine in screening the acidic surface sites of our low‐surface area samples.

The different modes of interaction of THF molecules with Lewis and Brönsted acid sites can be postulated by the same way as pyridine and dimethyl pyridine in many published papers[^40^, ^41^] as presented in scheme 1.

**Figure 5:**
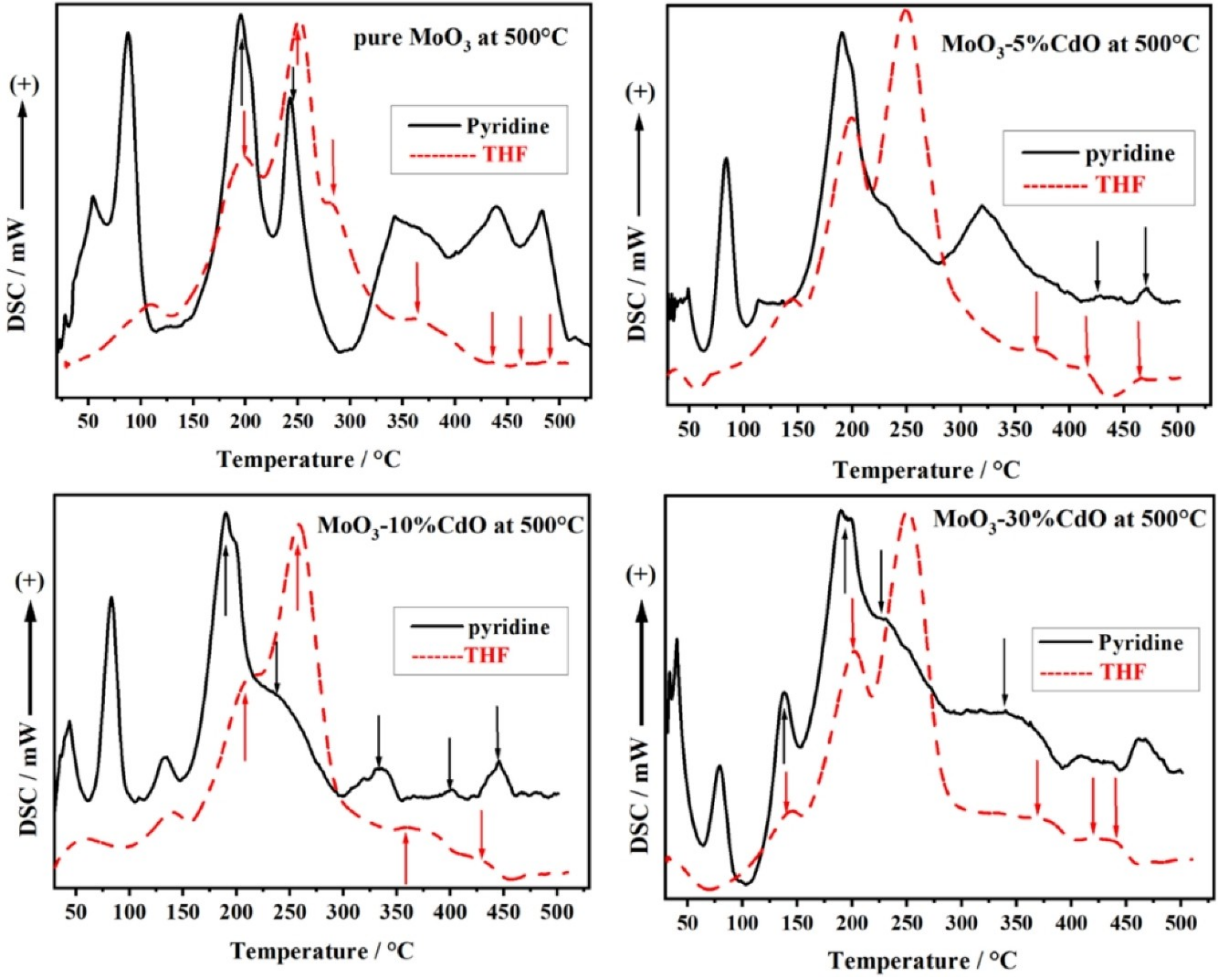
DSC‐TPD profiles recorded during desorption of pyridine (black) and THF (red) as probe molecules for MoO_3_ as pure or mixed with 5–30 % CdO calcined at 500 °C.

DSC‐TPD profiles of both pyridine and THF are shown in Figure 5 for all samples under study. There is a clear similarity between the two Pyr and THF profiles recorded for each sample, with a slight temperature shift of some peaks in the range of 125–450 °C. This shift can be ascribed to the physical nature of each molecule, as well as bond strength between the probe molecule and the acidic site, as will be discussed further below.

No further comments could be made to explain the similarity, completeness, and accuracy of using THF as a probe molecule. Despite their small surface areas, this smart molecule successfully screened all types of acidic sites in our samples, including weak, moderate and strong acidic sites. THF and pyridine DSC‐TPD profiles can provide a qualitative overview of the distribution of the acidic sites of the surface of our samples.

**Figure 6:**
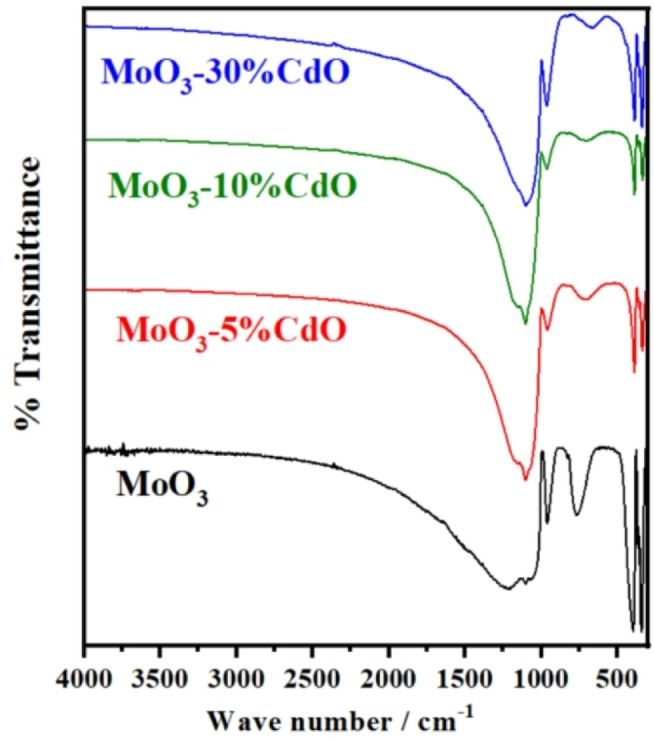
FTIR spectra of MoO_3_ as pure and mixed samples with 5–30 % CdO calcined at 500 °C for 5 h.

Recalling our DSC‐TPD results, as shown in Figure 5, for MoO_3_ as pure and mixed with 5–30 % CdO calcined at 500 °C, which confirm the existence of Lewis‐acidic surface sites only.. By examining all the FTIR spectra of our samples, as presented in Figure 6, one can notice that none of the absorption bands due to the presence of hydroxyl (O−H) stretching modes at ≈3400 cm^−1^ or at 1640 cm^−1^ that assigned to O−H bending vibrations^[42]^ of adsorbed water are recorded for these samples. This can be ascribed to the high calcination temperature for an extended period of time, that is, 500 °C and 5 h. Only the absorption bands at 966 cm^−1^ due to Mo=O vibrations,^[42]^ in the range 771–670 cm^−1^ of the stretching vibration of O−Mo‐O^[43]^ in [MoO_4_]^2−^ tetrahedron, and a weak band at 384 cm^−1^ for the bending vibration of Mo−O are present.^[43]^ Spectra of mixed samples exhibited two bands at 1170 and 1100 cm^−1^ attributed to the metal‐oxygen stretching of Cd−O in these mixed samples.^[44]^ The description mentioned above of FTIR spectra of our samples revealed that none of the hydroxyl groups or water molecules are chemically associated with the surface of these samples. As a result, THF molecules can only interact with the Lewis‐acidic cationic sites, as discussed later.

**Figure 7:**
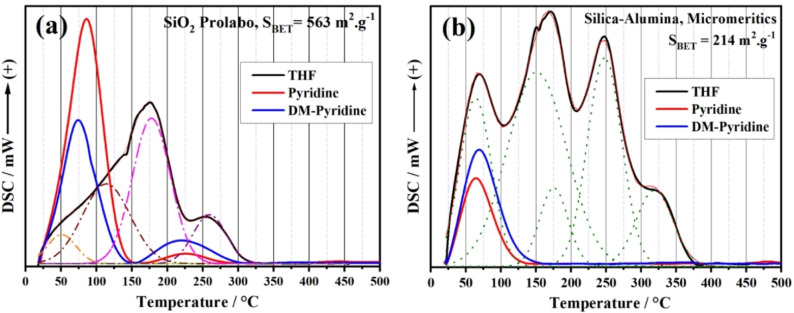
DSC‐TPD profiles recorded during desorption of pyridine, DM‐pyridine and THF as probe molecules for SiO_2_ (a) and silica‐alumina (b) as reference samples.

To support the results obtained for samples under investigation herein, we examined the validity of THF as a smart and novel probe molecule for assessing the acidic surface sites using TPD technique, compared with pyridine and dimethyl pyridine, two different and well‐known probe molecules. We employed the DSC‐TPD technique separately using each probe molecule of the SiO_2_ sample, purchased from Prolabo‐France with S_BET_=563 m^2^ ⋅ g^−1^, and another reference sample of silica‐alumina (Micromeritics‐USA) with S_BET_=214 m^2^ ⋅ g^−1^. Figures 7a,b show the DSC‐TPD profiles of both samples using THF, pyridine (Pyr) and dimethyl pyridine (DMPyr) as probe molecules. In the case of SiO_2_, Figure 7a, both Pyr and DMPyr gave two peaks, relatively coincides with each other, due to desorption of weakly physisorbed probe molecules followed by desorption of Pyr and DMPyr from Lewis and Brönsted acid sites of the SiO_2_ sample. On the other hand, the DSC‐TPD of THF showed a huge curve with different features. A deconvolution Gaussian line shape is demonstrated for a better and more accurate classification of these sites with different strengths under the THF DSC‐TPD profile, as seen in Figure 7a. This has resulted in the appearance of three types of acidic sites, that is, weak, medium and strong acidic sites, as well as the band of the weakly physisorbed THF molecules. In the case of the other sample, silica‐alumina, DSC‐TPD profiles showed only a single peak due to desorption of weakly physisorbed probe molecules of Pyr and DMPyr. Using these probe molecules, no desorption peaks were recorded for any type of acidic sites. On the contrary, the DSC‐TPD profile using THF as a probe molecule exhibited a fully descriptive picture of the acidic surface sites of this sample, see Figure 7b. Obviously, the deconvolution Gaussian line shape of this profile accurately classified these acidic sites as two peaks of weak sites, one peak of medium sites, and a single peak of strong acidic sites (see Figure 7b), as well as that peak of the physisorbed probe molecules of THF. Many published articles applied NH_3_‐TPD technique[^4^, ^5^, ^15^] to classify the strength of the acidic sites, while others measured the IR‐spectra of pyridine[^1^, ^3^, ^18^, ^20^] for the same purpose. Nothing was stated about quantitatively estimating the acidic sites with pyridine or using pyridine‐recorded DSC‐TPD profiles. Therefore, from the first glance at DSC‐TPD profiles, Figures 5 and 7 suggest that THF should be used as a smart probe molecule to screen the acidic surface sites accurately. The advantages of THF can be summarised as follows: (1) it easily interacts with both Lewis and Brönsted acid sites (see Scheme 1) to measure the total surface acidity of solid catalysts; (2) it clearly differentiates between the capacity of surface acidity of different samples as evidenced by the profiles of the reference samples (Figure 7); (3) it provides a full picture of the strength of these sites, regardless the samples have low or high surface area. THF′s inability to distinguish between Lewis and Brönsted acid sites may also be a disadvantage of this molecule. Using other techniques such as IR‐pyridine desorption measurements in conjunction with DSC‐TPD of THF, will provide a good estimate of the acid site types and strength.

### Dehydration of Isopropyl Alcohol (IPA)

Dehydration of IPA [Eq. (2)] was used as a probe reaction to examine the catalytic activity of four samples, namely pure MoO_3_ and mixed with 5, 10 and 30 mol % CdO (wt/wt), respectively, which yielded rational values during the TG‐TPD experiments of both Pyr and THF.

(2) $$\mathrm{CH}{}_{3}\text{-}\mathrm{CH}\left(\mathrm{OH}\right)-\mathrm{CH}{}_{3}\;\to \;\mathrm{CH}{}_{3}\text{-}\mathrm{CH}=\mathrm{CH}{}_{2}+H{}_{2}O$$

**Figure 8:**
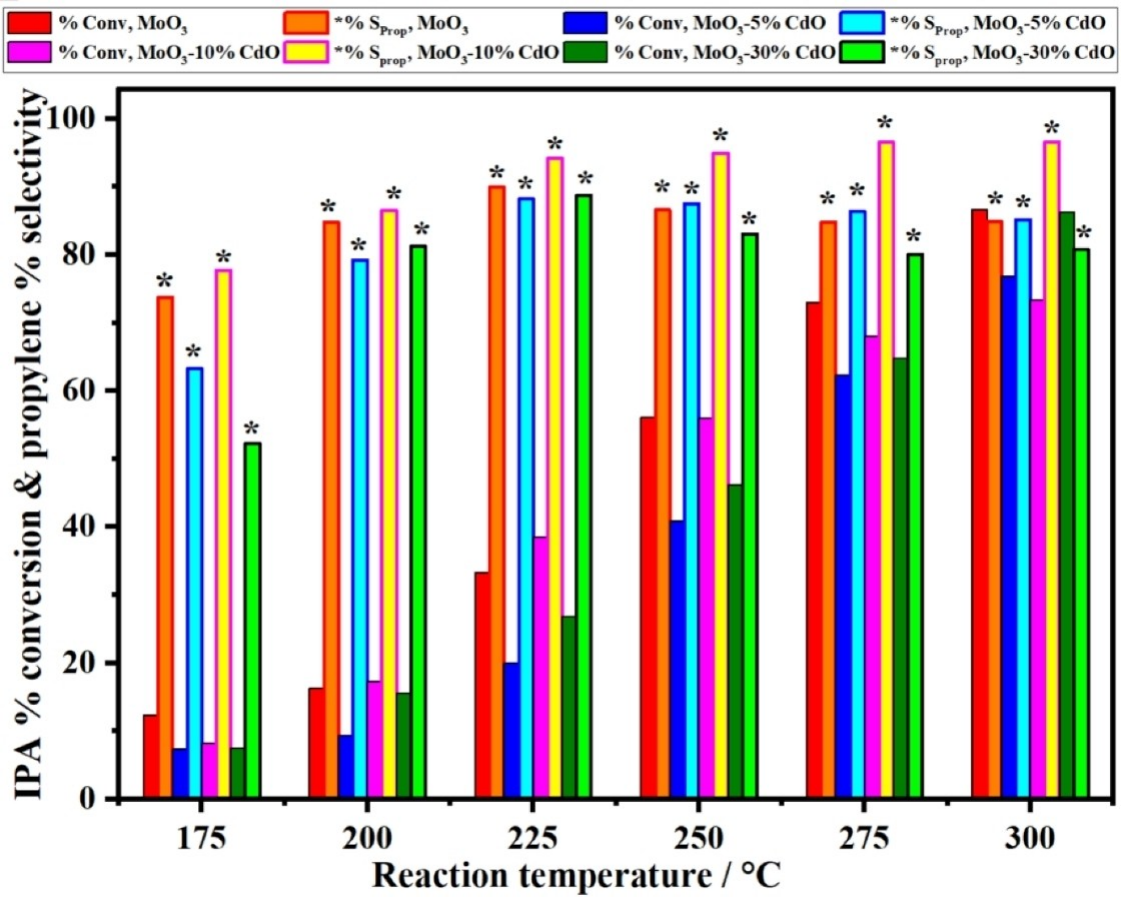
Dehydration reaction temperature of IPA as a function of % conversion and propylene selectivity, as the main product, over the specified catalysts in the temperature range of 175–300 °C.

Besides, to correlate their activities with the calculated number of acidic surface sites of such samples, as shown in Table 1, the dehydration reaction was carried out in the temperature range of 175–300 °C, as shown in Figure 8, where the main product was propylene with minimum selectivity of ≥80 % in a temperature range of 200–300 °C.

The following key points are easily apparent from the data illustrated in Figure 8:

1. At reaction temperatures of ≤200 °C, % conversion of IPA over all samples was in the range of 7–17 %. This can only be attributed to the contribution of weak acidic sites in this temperature range, as shown by the TG‐TPD curves in Figure 3.
2. Increasing the reaction temperature, that is, 225–300 °C, the % conversion of IPA to propylene steadily improved. At the corresponding reaction temperature, the reactivity of each sample slightly varied in comparison with other samples. This could be due to the different facets of both MoO_3_ and CdMoO_4_ hosted in MoO_3_ and exposed to IPA molecules^[18]^ during the dehydration reaction of IPA. Furthermore, the limited surface area of these samples is an important factor that reflects this discrepancy in their recorded reactivity.
3. The last clear point in this sequence, as shown in Figure 8, is that sample MoO_3_‐10 % CdO has the highest % selectivity of propylene production at all reaction temperatures among the other four samples. These findings are strongly supported by the calculated value of the total number of acidic sites of such sample using THF, which is shown in Table 1 and illustrated in Figure 4. On the other hand, were incompatible with the calculated number of acidic sites in total samples using TG‐TPD of pyridine. In a recently published paper,^[18]^ based on their findings, the authors suggested that infrared (IR) spectroscopy of Pyr can be used to qualitatively identify the different types of acidic sites, but it was unable to accurately determine these sites quantitatively. Therefore, the majority of the published articles concerning the acidity measurements of catalysts used IR spectroscopy of pyridine and TG and/or DSC TPD of NH_3_ to quantify total acidity.[^1^, ^3^, ^45^, ^46^] Our calculated results of the TG‐TPD experiments using THF, as shown in Table 1, were approximately 1.6‐2.7 times those calculated using TG‐TPD of pyridine over the four samples. The higher the population of the THF molecules over the sample surface, hence an accurate calculation of the total accessible acidic sites will be achieved. In this regard, THF may provide a valuable advantage as a probe molecule for quantifying the total acidic sites in our samples.

Due to the highly acidic properties of MoO_3_, it significantly enhances the acidity of other oxides such as TiO_2_, SnO_2_ ^[47]^ and SiO_2_.^[48]^ Some authors linked the activity of MoO_3_ in heterogeneous catalytic esterification reactions to the Lewis‐acidic sites,^[48]^ which are thought to be the main active sites of such material. Another research group^[49]^ proposed that the thermal treatment and the conditions used during the preparation of MoO_3_ drastically alter the population and types of acidic sites on its surface. Very recently, in a published article, high‐density Lewis acid sites were created on growing porous single‐crystalline Mo_2_N and MoN.^[50]^

## Results Discussion

Optimising analytical tools such as TG and DSC‐TPD experiments, particularly by attempting new probing molecules such as THF, can assist in determining the acidic sites of very low surface area catalysts. The TG‐TPD method provides a total quantitative number of the acidic surface sites of each sample, whereas the DSC‐TPD technique offers a qualitative distribution of the different types of these acidic sites. Therefore, the analysis tool used herein enables us successfully to compare the two different probe molecules, that is, pyridine as a widely used molecule and THF as a proposed new smart probe molecule. THF, which we choose in this study as a probe molecule, is a heterocyclic organic compound with high polarity as ether and low boiling point, that is, 66 °C,^[51]^ which offers a high vapour pressure at 20 °C about 8 times that of pyridine.^[51]^ Because of its higher value of vapour pressure of 17.6 kPa, this advantage can produce a large number of THF molecules in the vapour phase,^[51]^ which interact with the Lewis‐acidic cationic sites, that is, Mo‐atoms, of the MoO_3_ structure^[52]^ as we propose in the following Scheme 2 (I):

Furthermore, these THF molecules interact with the Lewis‐acidic cationic sites, that is, Mo and Cd atoms, of CdMoO_4_ in samples of MoO_3_ mixed with 5–30 % CdO as in Scheme 2(II). Scheme 2(II) appears to be similar to a recently proposed mechanism^[53]^ of the hydrogenolysis of dibenzofuran over Co/MoO_3_ catalyst via the acidic sites of such catalyst. Pyridine has a clear disadvantage compared to THF as a probe molecule due to its higher boiling point of 115.2 °C and low vapour pressure of 2.13 kPa.^[51]^ Finally, there is a clear agreement between all the recorded data of sample MoO_3_‐10 % CdO, which is characterised by the highest surface area values, the population of Lewis‐acidic sites and % selectivity of propylene at all the applied reaction temperatures. These findings support one another in demonstrating that THF, as a suitable and smart molecule, assesses the acidic surface sites of metal oxide catalysts, especially those with very low surface area.

According to the previously mentioned data and the proposed mechanisms, it is worthwhile to try testing THF as a probe molecule for quantitatively and quantitatively assessing the surface Lewis‐acidic sites of catalysts using TG and DSC‐TPD techniques in various catalytic materials, particularly those with very low surface area.

## Conclusion

Measuring the Lewis‐acidic surface sites in catalysis is problematic when the surface area of the material is very low; in this work, we measured it for the first‐time using tetrahydrofuran (THF) as a probe molecule in the vapour phase. Herein, we assessed the Lewis‐acidic surface sites of selected samples of MoO_3_ as pure and mixed with 5–30 % CdO (wt/wt), as well as CdO for comparison by applying TG and DSC temperature‐programmed desorption (TPD) techniques. Comparing the obtained results with another set of results recorded using pyridine as a widespread probe molecule in determining acidic sites using the same techniques showed a good agreement. This audition is based on the limited values of the surface area of these samples (i. e., very low S_BET_ ≤1 m^2^ ⋅ g^−1^) that likely require a relatively moderate basic molecule as THF with p*K*_b_=16.08, rather than strong basic molecules such as NH_3_ (p*K*_b_=4.75) or pyridine (p*K*_b_=8.77). Our calculated data in the case of THF are in good agreement with the determined values of the surface area of these samples, except CdO. There is a clear similarity between the DSC‐TPD curves recorded for THF and pyridine. For pure MoO_3_ and mixed MoO_3_‐CdO samples, we proposed mechanisms for the interaction of vapour phase molecules of THF with the Lewis cationic Mo and Cd atoms. In addition, dehydration of isopropyl alcohol (IPA) was used as a probe reaction to investigate the catalytic activity of MoO_3_ as pure and mixed with 5–30 % CdO (wt/wt), to further support our findings in the case of THF in the temperature range of 175–300 °C. There is clear conformity between the obtained data of sample MoO_3_‐10 % CdO, which is characterised by the highest surface area value, the population of Lewis‐acidic sites and % selectivity of propylene at all the applied reaction temperatures. These findings support one another in demonstrating that THF is a suitable and smart molecule for assessing the acidic surface sites of metal oxide catalysts.

## Disclaimer

The views and opinions expressed in this paper do not necessarily reflect those of the European Commission or the Special EU Programmes Body (SEUPB).

## Conflict of interest

The authors declare no conflict of interest.

1

## Data Availability

Data sharing is not applicable to this article as no new data were created or analyzed in this study.
